# Molecular cloning and analysis of zebrafish voltage-gated sodium channel beta subunit genes: implications for the evolution of electrical signaling in vertebrates

**DOI:** 10.1186/1471-2148-7-113

**Published:** 2007-07-10

**Authors:** Sameer S Chopra, Hiroshi Watanabe, Tao P Zhong, Dan M Roden

**Affiliations:** 1 Department of Pharmacology, Vanderbilt University School of Medicine, Nashville, TN, 37232, USA; 2 Cell & Developmental Biology, Vanderbilt University School of Medicine, Nashville, TN, 37232, USA; 3 Medicine, Vanderbilt University School of Medicine, Nashville, TN, 37232, USA

## Abstract

**Background:**

Action potential generation in excitable cells such as myocytes and neurons critically depends on voltage-gated sodium channels. In mammals, sodium channels exist as macromolecular complexes that include a pore-forming alpha subunit and 1 or more modulatory beta subunits. Although alpha subunit genes have been cloned from diverse metazoans including flies, jellyfish, and humans, beta subunits have not previously been identified in any non-mammalian species. To gain further insight into the evolution of electrical signaling in vertebrates, we investigated beta subunit genes in the teleost *Danio rerio *(zebrafish).

**Results:**

We identified and cloned single zebrafish gene homologs for beta1-beta3 (*zbeta1-zbeta3*) and duplicate genes for beta4 (*zbeta4.1, zbeta4.2*). Sodium channel beta subunit loci are similarly organized in fish and mammalian genomes. Unlike their mammalian counterparts, *zbeta1 *and *zbeta2 *subunit genes display extensive alternative splicing. Zebrafish beta subunit genes and their splice variants are differentially-expressed in excitable tissues, indicating tissue-specific regulation of *zbeta1-4 *expression and splicing. Co-expression of the genes encoding zbeta1 and the zebrafish sodium channel alpha subunit Na_v_1.5 in Chinese Hamster Ovary cells increased sodium current and altered channel gating, demonstrating functional interactions between zebrafish alpha and beta subunits. Analysis of the synteny and phylogeny of mammalian, teleost, amphibian, and avian beta subunit and related genes indicated that all extant vertebrate beta subunits are orthologous, that beta2/beta4 and beta1/beta3 share common ancestry, and that beta subunits are closely related to other proteins sharing the V-type immunoglobulin domain structure. Vertebrate sodium channel beta subunit genes were not identified in the genomes of invertebrate chordates and are unrelated to known subunits of the *para *sodium channel in *Drosophila*.

**Conclusion:**

The identification of conserved orthologs to all 4 voltage-gated sodium channel beta subunit genes in zebrafish and the lack of evidence for beta subunit genes in invertebrate chordates together indicate that this gene family emerged early in vertebrate evolution, prior to the divergence of teleosts and tetrapods. The evolutionary history of sodium channel beta subunits suggests that these genes may have played a key role in the diversification and specialization of electrical signaling in early vertebrates.

## Background

Coordinated electrical signals in the metazoan nervous system, heart, and skeletal muscle depend on the generation of action potentials, rapid changes in membrane potential mediated by the passage of ions through voltage-gated ion channels [1]. The initial upstroke of the action potential in most excitable cells is determined by sodium channels, membrane proteins characterized by rapid activation and inactivation and high ionic conductance [1-3]. Unlike evolutionarily-ancient, tetrameric potassium channels, sodium channels are comprised of four homologous domains that are encoded by a single polypeptide [1-3]. Sodium channels are suspected to have evolved from structurally-similar calcium channels, which in turn likely arose following the duplication of potassium channel genes [1-3]. The use of sodium as a conducting ion rather than calcium is thought to have permitted rapid conduction and high-frequency electrical signaling in the rudimentary nervous systems of early multicellular animals, without the numerous additional intracellular effects mediated by calcium's role as a second messenger [1,4].

In mammals, voltage-gated sodium channels are multi-protein complexes that include a pore-forming α subunit and 1 or more modulatory β subunits [5-9]. While sodium channel α subunits are large proteins with 24 membrane-spanning domains, β subunits are smaller proteins with a single transmembrane domain and an extracellular V-type immunoglobulin (IG)-like motif [10-14]. Ten distinct α subunit genes (*SCNxA*) and 4 β subunit genes (*SCN1B-4B*) have been cloned from mammals, and all have been functionally expressed with the exception of *SCN7A *[2,3,15]. While heterologous expression of α subunit genes alone can reconstitute key properties of voltage-gated sodium channels observed in native tissues, co-expression with β subunit genes modifies channel gating and increases channel cell surface expression [10-13]. Studies of mice with targeted ablation of either β1 or β2 genes corroborated these roles for β subunits *in vivo *and revealed an additional function for β subunits in α subunit localization [16,17]. Moreover, the unique expression profiles of each β subunit gene and their variable effects on α subunit function, expression, and localization suggest that different subunit combinations in heart, brain, and muscle may contribute to diversity and specialization in electrical signaling [18,19].

The physiological importance of sodium channel α and β subunits is reinforced by their role in human disease. Mutations that cause even subtle changes in sodium channel α subunit function may result in severe human disease phenotypes including epilepsy and arrhythmia [20]. To date, mutations in 7 α subunit genes (*SCN1A, SCN2A, SCN3A, SCN4A, SCN5A, SCN8A, SCN9A*) have been linked to human disease [21-27]. Given their important modulatory effects on the expression and function of sodium channel α subunits, β subunits are also important candidate genes for human diseases related to sodium channel dysfunction. β1 subunit gene mutations are a cause of generalized epilepsy with febrile seizures plus (GEFS+), and variants in sodium channel α subunits that disrupt their interaction with β subunits may be pathological in the heart [28-31]. More recently, a mutation in the β4 subunit gene was implicated as a cause of the Long QT syndrome, a congenital arrhythmia [32].

The relatively large number of distinct mammalian sodium channel α subunit isoforms has generated great interest in the evolution of this gene family, particularly since invertebrate genomes contain far fewer conserved Na_v_1 sodium channel genes [33-36]. The physical linkage of voltage-gated sodium channel genes to the 4 homeobox (HOX) gene clusters suggests that polyploidization at least partly contributed to the emergence of multiple ancestral sodium channel genes in early vertebrates [37-39]. Moreover, the clustering of closely-related sodium channel genes in mammalian genomes (e.g.* SCN1A*, *SCN2A*, and *SCN3A* on human chromosome 2) is consistent with the hypothesis that several of the 10 mammalian isoforms arose following tandem duplication of ancestral vertebrate genes [36-39]. Since fish and mammals possess different numbers of sodium channel isoforms, the duplication of ancestral vertebrate sodium channel genes is suspected to have occurred independently in teleost and tetrapod lineages [38]. Expansion of the sodium channel gene family by tandem duplication may also have occurred uniquely in tetrapods, since the complement of sodium channel genes in teleosts appears to have resulted from whole-genome duplication [40,41].

The unique electrophysiological properties, expression patterns, and physiological roles of mammalian sodium channel α subunits suggest that the duplication of these genes may have been an adaptation that permitted the diversification and functional specialization of electrical signaling in vertebrates [1,39,42]. The physical interaction of α subunits with auxiliary proteins such as β subunits that modify their expression or function may have been similarly adaptive [36,37]. Although sodium channel β subunits have not previously been identified in non-mammalian species, we hypothesize that a sodium channel macromolecular complex comprised of α subunits and auxiliary β subunits is an evolutionarily-conserved structural entity. Evidence for the emergence of sodium channel β subunit genes early in vertebrate evolution would be consistent with the idea that these genes may have played a role in diversifying and fine-tuning electrical signaling not only in mammals but in all vertebrates.

Here we report the identification and molecular cloning of conserved orthologs of all 4 mammalian β subunit genes in *Danio rerio *(zebrafish), a teleost vertebrate and a popular developmental and physiological model system. By direct sequence analysis and alignment, we assessed whether zebrafish β subunits possess the structural hallmarks of their mammalian counterparts. We provide evidence for extensive splicing of zebrafish beta subunit genes and demonstrate their expression in excitable tissues. To determine whether zebrafish sodium channel α and β subunits functionally interact, we studied sodium currents generated by the co-expression of the genes encoding zNa_v_1.5 (α subunit) and zβ1 in a heterologous cell system. Finally, we assessed the synteny and phylogeny of β subunits and a group of closely-related genes with IG domains in several vertebrate species. Our findings strongly suggest that all 4 voltage-gated sodium channel β subunit genes emerged early in vertebrate evolution, and support the concept that the voltage-gated sodium channel α-β subunit macromolecular complex is a conserved and functionally-important vertebrate innovation.

## Results

### Identification and cloning of zebrafish sodium channel β subunit genes

**Figure 1:**
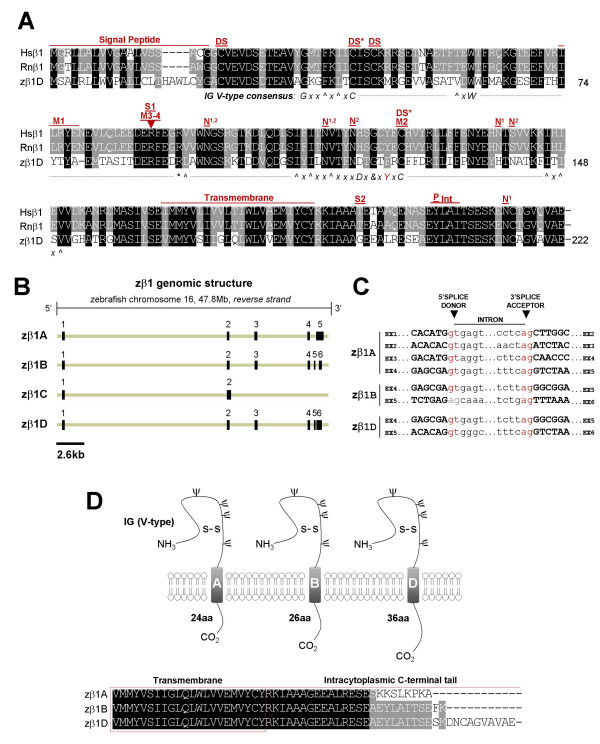
**Analysis of the cloned zebrafish β1 subunit gene and novel splice variants**. *A) *Alignment of cloned human, rat, and zebrafish β1 amino acid sequences. Black = identical in all three species; grey = identical in 2/3 species or conserved substitution. Shown for zebrafish is the most conserved β1 splice form (variant D). Hs = *Homo sapiens*, Rn = *Rattus norvegicus*, z = zebrafish; DS* = cysteine residue predicted to participate in a disulfide bridge, based on the myelin P0 protein crystal structure; DS = predicted second disulfide bridge; N = predicted N-linked glycosylation site (N1 = human/rat, N2 = zebrafish); M1 = site of epilepsy-causing deletion (I70_E74del) in Hsβ1; M2 = site of second epilepsy-causing mutation (C121W) in Hsβ1; M3/4 = site of third and fourth epilepsy-causing mutations (R85C, R85H); S1 = nonsynonymous Hsβ1 single nucleotide polymorphism (SNP, G/A > R85H); S2 = nonsynonymous Hsβ1 SNP (C/T > T189M); P = phosphorylation site (tyrosine Y181) that regulates ankyrin recruitment (NOTE: Y200 = Y181 following cleavage of 19 amino acid signal peptide); IN = putative internalization sequence. Consensus sequence for V-type IG domain is depicted beneath the alignment: G = glycine, x = any residue, ^ = hydrophobic residues, C = cysteine, - = gap in alignment with consensus sequence, W = tryptophan, * = basic residue, L = leucine, D = aspartic acid, & = glycine, alanine, or aspartate, and Y = tyrosine. Red indicates zebrafish residues that deviate from the consensus sequence. See Results for references supporting sequence annotation. *B) *5' and 3' RLM-RACE PCR and RT-PCR identified four distinct splice variants expressed from zβ1 locus on zebrafish chromosome 16 (*Ensembl*). *C*) Splice donor and acceptor sites of zebrafish β1 splice variants, derived from comparing cloned cDNA against genomic DNA sequences (*Ensembl*). Consensus GT-AG splice sites are labeled in red. A splice-site deviating from the consensus appears in grey. *D*) Schematic diagram of β1 splice variants A, B, and D, whose predicted proteins differ only in the length of their intracytoplasmic C-terminal tail. S-S = disulfide bridge. NH_3 _= 5' amino terminus, CO_2 _= 3' carboxyl terminus, β = putative N-linked glyosylation site. Alignment of C-terminal tail of variants zβ1A, zβ1B, and zβ1D (below). zβ1C is not shown as it is predicted to lack both extracellular IG and transmembrane domains.

**Figure 2:**
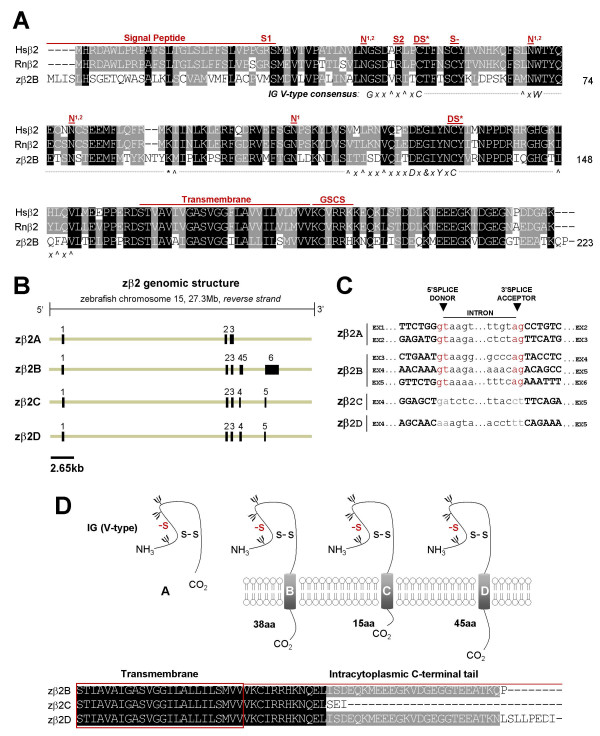
**Analysis of the cloned zebrafish β2 subunit gene and novel splice variants**. Presentation and labeling as in Figure 1. *A) *Alignment of cloned human, rat, and zebrafish β2 amino acid sequences. Shown for zebrafish is the most conserved β2 splice form (*variant B*). S- = conserved cysteine in β2 that is a putative site of covalent linkage with a partner α subunit; S1 = nonsynonymous Hsβ2 single nucleotide polymorphism (SNP, C/T > R28W) (NCBI dbSNP, PharmGKB); S2 = nonsynonymous Hsβ2 SNP (G/A > R47H) (NCBI dbSNP, PharmGKB); GSCS = γ-secretase cleavage site. *B) *5' and 3' RLM-RACE PCR and RT-PCR identified four distinct splice variants expressed from the zβ2 locus on zebrafish chromosome 15 (*Ensembl*). *C) *Splice donor and acceptor sites of zebrafish β2 splice variants. Zβ2 variants C and D both differ from the consensus sequences at the exon 4-intron 4 and intron 4-exon 5 splice junctions. *D) *Schematic diagram of β2 splice variants A-D. With the exception of variant A, the predicted proteins of zβ2 variants B-D differ only in the length of their intracytoplasmic C-terminal tail. Alignment of C-terminal tail of variants zβ2B, zβ2C, and zβ2D (below).

**Figure 3:**
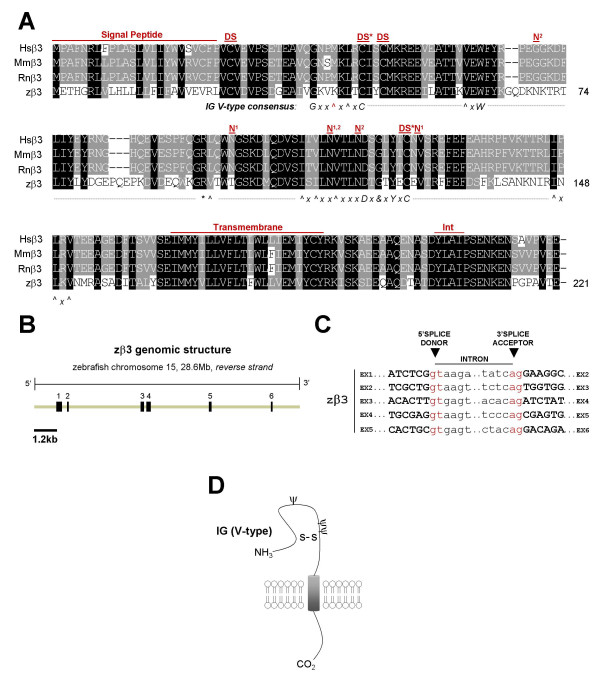
**Analysis of the cloned zebrafish β3 subunit gene**. Presentation and labeling as in Figure 1. *A) *Alignment of cloned human, mouse, rat, and zebrafish β3 amino acid sequences. INT = putative internalization sequence. *B) *5' and 3' RLM-RACE PCR and RT-PCR identified a single transcript expressed from the zβ3 locus on zebrafish chromosome 15 (*Ensembl*). zβ3 has six exons. *C) *All zβ3 splice sites adhere to GT-AG consensus sequences. *D) *Schematic diagram of the β3 protein.

**Figure 4:**
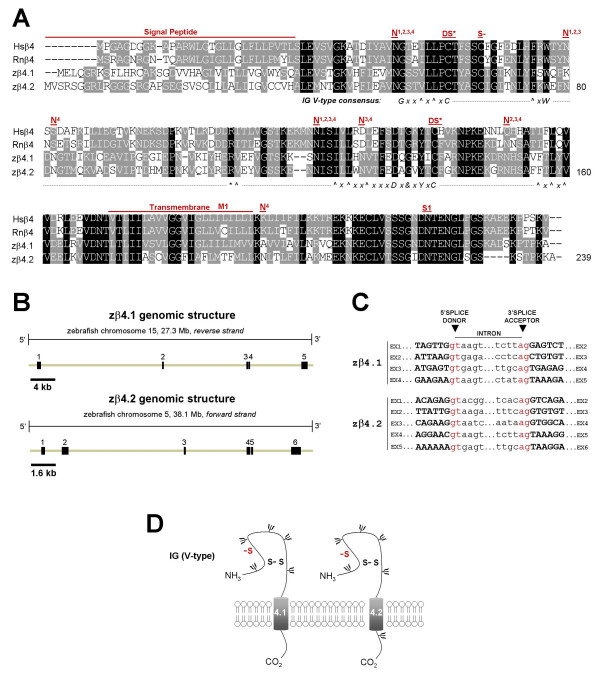
**Analysis of cloned zebrafish β4.1 and β4.2 subunit genes**. Presentation and labeling as in Figure 1. *A) *Alignment of cloned human, rat, and zebrafish β4 amino acid sequences. S- = conserved cysteine in β4 that is a putative site of covalent linkage with a partner α subunit; N = predicted N-linked glycosylation site (N1 = human, N2 = rat, N3 = zebrafish β4.1, N4 = zebrafish β4.2); M = site of putative Long QT syndrome-causing mutation L179F; S1 = nonsynonymous Hsβ4 SNP (A/C > N210H). *B) *Genomic organization of zβ4.1 and zβ4.2 derived from comparing cloned cDNA sequences with genomic sequences of zebrafish chromosomes 15 and 5, respectively. zβ4.1 has five exons and zβ4.2 has six exons. *C) *All zβ4.1 and zβ4.2 splice sites exhibit consensus GT-AG donor/acceptor sequences. *D) *Schematic diagram of zβ4.1 and zβ4.2 proteins.

We identified 5 distinct zebrafish β subunit gene loci by mining the draft zebrafish genome sequence (ensembl Zv2-4) for genes homologous to the human and mouse β1- β4 genes. *In silico *prediction of coding sequence on the relevant genomic DNA contigs enabled us to amplify partial β subunit gene sequences for each locus by RT-PCR and subsequently complete the full-length zebrafish β subunit clones by rapid amplification of cDNA ends (RACE)-PCR. Analysis of putative open reading frames within each cDNA sequence predicted proteins ranging from 220–232 amino acids in length. Deduced translation and alignment of the five zebrafish amino acid sequences with each mammalian β subunit protein sequence suggested the identities of these genes (Figs. 1A, 2A, 3A, 4A). While single homologs were identified for the genes encoding β1-β3 (*zβ1-zβ3*), zebrafish express two distinct but closely-related β4 genes (*zβ4.1, zβ4.2*). Alignment of the amino acid sequences of zebrafish β1, β2, β3, β4.1, and β4.2 with their human homologs indicated that these proteins share 53.4%, 49.6%, 51.1%, 43.2%, and 40.8% identity, respectively.

### Conserved features of zebrafish sodium channel β subunits

Mammalian sodium channel β subunits possess a single transmembrane domain, a short intracellular carboxyl C-terminal tail, a cleavable amine (N)-terminal signal peptide, and a conserved V-type extracellular IG motif that most closely resembles the type found in cell-adhesion molecules [14,43]. Hydropathy analysis of zebrafish β subunit amino acid sequences using TMpred software revealed that all 5 proteins are likely to possess cleavable N-terminal signal peptides and single transmembrane domains (additional file 1). To assess the presence of an extracellular V-type IG domain, we first analyzed each zebrafish beta subunit amino acid sequence with the NCBI conserved domain database. By this method, all 5 zebrafish β subunit genes are predicted to possess V-type extracellular IG domains (additional file 2). To more rigorously analyze these predictions, we manually examined the amino acid sequence of each zebrafish β subunit for the V-type IG domain consensus sequence (annotated beneath alignments, Figs. 1A, 2A, 3A, 4A). With the exception of a conservative tyrosine to phenylalanine (Y > F) substitution in zβ1 and a non-polar methionine to basic lysine residue substitution (M > K) in zβ3 (illustrated in red text in Figs. 1A and 3A), all 5 zebrafish β subunits exhibit 100% conservation of the consensus sequence for V-type IG domains [14,43]. Typical of this domain is the linkage of 2 cysteine residues in a disulfide bridge (DS*, Figs. 1A, 2A, 3A, 4A), the structural importance of which was revealed by a mutation that results in familial epilepsy [28]. A second putative disulfide bridge that may further stabilize the extracellular IG domain in β1 and β3 is suggested of the crystal structure of myelin protein zero (MPZ), the primary structural protein of peripheral nerve myelin, whose IG domain closely resembles that found in sodium channel β subunits [12,43,44]. The cysteine residues that contribute to proposed second bridge are conserved in zβ1 and zβ3 (DS, Figs. 1A, 3A). Similar to mammalian β2 and β4, zβ2 and zβ4 proteins do not possess this second disulfide bridge but have an unpaired cysteine residue that may underlie covalent interactions with partnering sodium channel α subunits (S-, Figs. 2A, 4A) [5,11,13].

In addition to V-type IG domains, our sequence analysis indicated that other functional and/or structural domains are conserved in zebrafish β subunits (Figs. 1A, 2A, 3A, 4A). These include a tyrosine in β1 that is phosphorylated and regulates interactions with ankyrin G (Y181 or Y200 with signal peptide intact) [45], the C-terminal tyrosine-leucine-alanine-isoleucine (Y-L-A-I) internalization motif in β1 and β3 which may be recognized by clathrin-coated pits [12], and a juxtatransmembrane gamma-secretase cleavage site in β2 that may play a role in cell adhesion or the movement of cells expressing this subunit [46]. Zebrafish β subunits are also likely to exhibit post-translational modification by amide nitrogen (N)-linked glycosylation, a key feature of all 4 mammalian β subunits [10-13]. The zβ1 protein possesses 2 of 4 predicted N-linked glycosylation sites found in human or rat β1 in addition to exhibiting two unique sites (Fig. 1A). Similarly, zβ2 conserves 3 of 4 sites, zβ3 conserves 1 of 3 sites and displays 2 novel sites, zβ4.1 conserves 4 sites and displays 1 novel site, and zβ4.2 conserves 3 of four sites and displays 3 novel sites (Figs. 2A, 3A, 4A).

### Comparative genomics of β subunit gene variants

The conservation of amino acid residues between homologous genes in distantly-related species may uncover previously unappreciated regions of functional importance and facilitate the evaluation of novel human mutations and polymorphisms. To determine whether previously identified mutations and polymorphisms are conserved in zebrafish β subunit genes, we mapped these variants onto the alignments of mammalian and zebrafish β subunit amino acid sequences. Four mutations in the human β1 gene have been linked to the heritable epilepsy syndrome GEFS+, the first resulting in a 5 amino acid deletion from isoleucine at position 70 to glutamic acid at position 74 (I70_E74del), the second in substitution of a cysteine residue that participates in a disulfide bridge (C121W), and the third and fourth resulting in substitution of an arginine at position 85 for either a cysteine or a histidine, respectively (R85C, R85H) [28,47,48]. All of these mutations are expected to destabilize the extracellular IG domain of β1 and result in loss of function. In zβ1, 2 of 5 amino acids in the deleted segment (isoleucine, tyrosine), C121, and R85 are all conserved (M1, M2, M3/4, Fig. 1A). R85H was also previously reported as a non-synonymous SNP in NCBI and PharmGKB SNP databases (S1, Fig. 1A). A second reported SNP in human β1 results in a threonine to methionine substitution at position 189 (T189M), but T189 is not conserved in the amino acid sequence of zβ1 (S2, Fig. 1A).

Although disease-causing mutations are not currently associated with either *SCN2B *or *SCN3B*, nonsynonymous SNPs have been reported for *SCN2B *that result in an arginine to tryptophan substitution at position 28 (R28W) and an arginine to histidine substitution at position 47 (R47H). R47 but not R28 is conserved in the amino acid sequence of zebrafish β2 (S1, S2, Fig. 2A). A mutation in *SCN4B *resulting in a leucine to phenylalanine substitution at position 179 (L179F) was recently implicated as a putative cause of the congenital Long QT syndrome [32]. Although this leucine is conserved in zebrafish β4.1, rat β4 and zebrafish β4.2 display a cysteine (C) and threonine (T) at this position, respectively (M1, Fig. 4A). A nonsynonymous *SCN4B *polymorphism has also been reported, resulting in an asparagine to histidine substitution at position 210 (N210H). N210 is conserved in the sequences of both zβ4.1 and zβ4.2 (S1, Fig 4A).

### Alternative splicing of zebrafish β subunit genes

The mammalian β1 subunit gene is alternatively-spliced in both rats (β1A) and humans (β1B), with retention of intron 3 being the primary event in both species that dramatically alters the transmembrane domain and intracellular C-terminus of the β1 subunit protein [49,50]. An alternative splice-variant of β1 that retains intron 5 and adds 86 nucleotides to the 3' untranslated region (UTR) of β1 mRNA transcripts has also been reported [51,52]. In zebrafish, we detected 4 alternatively-spliced variants of the β1 gene, designated here as *A-D*. Comparisons between complementary DNA and genomic DNA revealed that zβ1A transcripts have 5 exons while zβ1B and zβ1D each have 6 exons and zβ1C has only 2 (Fig. 1B, Table 1). Transcripts of zβ1A, zβ1B, and zβ1D all share exons 1 through 4 but not exons 5 and 6; and all splice junctions except exon 5-intron 5 of zβ1B display canonical GT-AG splice donor-acceptor sites (Figs. 1B, 1C; Table 1). The zβ1 splice variant that shares the most identity with human β1 at the amino acid level (zβ1D) also shares nearly identical genomic organization (Table 1). With the exception of differences in exons 5 and 6, zβ1A and zβ1B variants also share this conserved genomic architecture (Table 1).

**Table 1:** Comparative genomics of the sodium channel β1 gene in zebrafish and mammals.

**gene**	**Genbank accession number**	**gene location**	**cDNA**	**exon1**	**exon2**	**exon 3**	**exon 4**	**exon 5**	**exon6**	**protein**	**%ID (%SIM)**	**topology**
**zβ1A**	DQ489722	Chr 16	1411	219 (52)	167	238	142	645 (31)	-	209	47.5% (57.0%)	IG(V): 37–149 TM: 164–185
**zβ1B**	DQ489723	Chr 16	1054	219 (52)	167	238	142	28	260 (9)	211	49.8% (59.6%)	IG(V): 37–149 TM: 164–185
**zβ1C**	DQ489724	Chr 16	574	219 (52)	355 (266)	-	-	-	-	105	13.0% (17.5%)	IG(V): none TM: none
**zβ1D**	DQ489725	Chr 16	1320	219 (52)	167	238	142	72 (67)	482 (0)	221	53.4% (63.7%)	IG(V): 37–149 TM: 164–185
**hβ1**	NM_001037	Chr 19	1521	231 (40)	167	241	142	72 (67)	668 (0)	218	100% (100%)	IG(V): 33–146 TM: 161–182
**rβ1A**	AF182949	Chr 1	850	40	167	643 (615)	-	-	-	273	55.1% (59.1%)	IG(V): 33–146 TM: 216–234
**hβ1B**	NM_199037	Chr 19	1170	231 (40)	167	772 (600)	-	-	-	268	58.0% (60.2%)	IG(V): 33–146 TM: 244–263

Gene: z = zebrafish, h = human, r = rat, and letters A-D refer to different splice variants. Accession numbers are identifiers for nucleotide sequences in NCBI GenBank. Values for cDNA and exons represent number of nucleotides. (Parentheses) indicate nucleotides within the open reading frame (difference = untranslated sequence or UTR). Protein values represent amino acid number with signal peptide intact. %ID (%SIM) refers to percentage identity and similarity with the human β1 protein sequence as determined by alignment. Zebrafish β subunit topology was determined as described in Methods. IG(V) = V-type immunoglobulin domain and TM = transmembrane domain. Numbering refers to β1 protein sequence with signal peptide intact. IG(V) domain and transmembrane regions for human β1 and human/rat splice variants were annotated based on previous reports [10, 14, 43, 49, 50].

Similar to splice variants of the mammalian β1 gene, splice variants of zβ1 are all predicted to encode proteins with variable C-termini. Unlike the mammalian variants, however, zβ1A, zβ1B, and zβ1D are predicted to possess identical transmembrane domains and differ only in their distal C-termini (Fig. 1D). As a result of alternative-splicing, zβ1A lacks the conserved tyrosine residue (Y200, Fig. 1A) that was found to regulate recruitment of ankyrin G by mammalian β1, as well as the putative internalization sequence (Y-L-A-I) discussed above (Figs. 1A, 1D) [12,45,53]. These alterations may affect interactions between zβ1A and zebrafish ankyrin, as well as influence the cycling of the mature zβ1A protein from its membrane compartment. The zβ1C splice variant, which results from retention of intron 2 and has an open reading frame of only 105 amino acids, is predicted to lack both an extracellular IG motif and a membrane-spanning segment.

Although alternative splicing of the β2 gene has not previously been identified in mammals, we identified 4 unique β2 transcripts in zebrafish. While zβ2A and zβ2B are spliced at canonical splice donor and acceptor sites, variants zβ2C and zβ2D are assembled by splicing at non-canonical exon 4-intron 4 and intron 4-exon 5 splice donor and acceptor sites. zβ2A is assembled from only 3 exons, zβ2B from 6 exons, and zβ2C and zβ2D from 5 exons each (Fig. 2B). Mammalian and zebrafish β2 genes all share a second exon that is 167 nucleotides in length and encodes the initial segment of the subunit's extracellular IG domain. Otherwise, the genomic organization of zβ2 splice variants diverges from that of human, mouse, and rat β2 genes which all have 4 coding exons (Table 2). Nevertheless, exons 1 and 3 are similar in length in all species, as is the coding segment of exon 4 in the mammalian β2 genes and the zβ2B and zβ2D splice variants (Table 2). zβ2B, zβ2C, and zβ2D are unique in possessing a fifth exon that contributes to the gene's open reading frame. Similar to mouse β2, the most conserved zebrafish β2 splice variant at the amino acid level (zβ2B) has a lengthy terminal exon that is entirely non-coding.

**Table 2:** Comparative genomics of the sodium channel β2 gene in zebrafish and mammals.

**gene**	**Genbank accession number**	**gene location**	**cDNA**	**exon 1**	**exon 2**	**exon 3**	**exon 4**	**exon 5**	**exon 6**	**protein**	**%ID (%SIM)**	**topology**
**zβ2A**	DQ489726	Chr 15	821	243 (82)	167	411 (234)	-	-	-	160	32.6% (42.5%)	IG(V): 47–152 TM: none
**zβ2B**	DQ489727	Chr 15	2449	243 (82)	167	217	196	68 (10)	1558 (0)	223	49.6% (64.7%)	IG(V): 47–152 TM: 162–186
**zβ2C**	DQ489728	Chr 15	851	243 (82)	167	217	124	100 (13)	-	201	45.2% (58.4%)	IG(V): 47–152 TM: 162–186
**zβ2D**	DQ489729	Chr 15	918	243 (82)	167	217	193	98 (34)	-	231	48.1% (62.8%)	IG(V): 47–152 TM: 162–186
**Hsβ2**	NM_004588	Chr 11	4939	260 (70)	167	211	4301 (200)	-	-	215	100% (100%)	IG(V): 43–146 TM: 156–180
**Mmβ2**	NM_001014761	Chr 9	3980	213 (70)	167	211	618 (200)	2771 (0)	-	215	92.1% (94.4%)	IG(V): 43–146 TM: 156–180
**Rnβ2**	NM_012877	Chr 8	873	236 (70)	167	211	259 (200)	-	-	215	93.0% (94.9%)	IG(V): 43–146 TM: 156–180

Presentation and labeling as in Table 1. %ID (%SIM) refers to percentage identity and similarity with the human β2 protein sequence as determined by alignment. Zebrafish β2 subunit topology was determined as described in Methods. Numbering refers to β2 protein sequence with signal peptide intact. IG(V) domain and transmembrane regions for human β2 and human/mouse/rat splice variants were annotated based on previous reports [11, 14, 43, 101].

As observed for zβ1, the conceptual translation of zβ2 splice variants predict zβ2 proteins that are of variable length and amino acid sequence at their intracellular C-terminal tail (Fig. 2D). The variant zβ2A results from retention of intron 3 and is predicted to result in a 160 amino acid protein that has an intact extracellular IG domain but no transmembrane domain (Fig. 2D). zβ2A is thus unlikely to display canonical beta subunit activities such as modulation of the trafficking and/or function of voltage-gated sodium channels α subunits, or mediation of cell-adhesion between cells expressing this protein and other cells or the extracellular matrix, which all depend on integration in the cell membrane. Transcripts for variants zβ2B-D result from alternative splicing of exons 4–6 and are expected to produce proteins of 223, 201, and 231 amino acids, respectively (Fig. 2D, Table 2). Since the function of the intracellular C-terminal tail of zβ2 is not well-characterized, the predicted impact of these splice variants on zβ2 function cannot be readily predicted.

Unlike the zβ1 and zβ2 genes, alternatively-spliced transcripts of zβ3, zβ4.1, and zβ4.2 genes were not detected. For each of these three genes, the genomic organization is well-conserved compared to that of its respective mammalian homolog (Tables 3, 4). The open reading frame of *SCN3B *in humans, mice, rats, and zebrafish is derived from 5 exons (exons 2–6), with a non-coding initial exon found in all 4 species and a non-coding terminal exon found only in the mammalian β3 gene (Table 3). Splicing of zebrafish β3 is determined by canonical GT-AG splice donor and acceptor sites, producing a putative protein whose secondary structure is similar to the β3 subunit found in mammals (Figs. 3C, D). z*β4.1 *and *zβ4.2 *transcripts are comprised of 5 and 6 exons, respectively, all of which are assembled from splicing at canonical GT-AG splice donor and acceptor sites (Fig. 4B, C). *zβ4.2 *is unique among β4 genes in possessing a non-coding exon 1 (Table 4). The predicted secondary structures of zβ4.1 and zβ4.2 are both similar to the β4 subunit found in mammals (Fig. 4D).

**Table 3:** Comparative genomics of the sodium channel β3 gene in zebrafish and mammals.

**gene**	**Genbank accession number**	**gene location**	**cDNA**	**exon 1**	**exon 2**	**exon 3**	**exon 4**	**exon 5**	**exon 6**	**exon 7**	**protein**	**%ID (%SIM)**	**topology**
**zβ3**	DQ489730	Chr 15	1006	294 (0)	103 (55)	170	235	139	65 (64)	-	220	51.1% (65.2%)	IG(V): 38–150 TM: 165–186
**Hsβ3**	NM_018400	Chr 11	4052	778 (0)	80 (55)	164	226	139	86 (64)	2579 (0)	215	100% (100%)	IG(V): 38–145 TM: 160–181
**Mmβ3**	NM_178227	Chr 9	3549	206 (0)	80 (55)	164	226	139	80 (64)	2654 (0)	215	97.7% (97.7%)	IG(V): 38–145 TM: 160–181
**Rnβ3**	NM_139097	Chr 8	3910	350 (0)	82 (55)	164	226	139	80 (64)	2837 (0)	215	98.1% (98.1%)	IG(V): 38–145 TM: 160–181

Presentation and labeling as in Table 1. %ID (%SIM) refers to percentage identity and similarity with the human β3 protein sequence as determined by alignment. Zebrafish β3 subunit topology was determined as described in Methods. Numbering refers to β3 protein sequence with signal peptide intact. IG(V) domain and transmembrane regions for human β3 and human/mouse/rat splice variants were annotated based on previous reports [12, 14].

**Table 4:** Comparative genomics of the sodium channel β4 gene in zebrafish and mammals.

**gene**	**Genbank accession number**	**gene location**	**cDNA**	**exon -1**	**exon 1**	**exon 2**	**exon 3**	**exon 4**	**exon 5**	**protein**	**%ID (%SIM)**	**topology**
**zβ4.1**	DQ489731	Chr 22	2072	-	597 (91)	164	220	130	961 (94)	232	43.2% (59.3%)	IG(V): 53–155 TM: 165–187
**zβ4.2**	DQ489732	Chr 5	1490	233 (0)	410 (97)	164	223	130	330 (85)	232	40.8% (56.7%)	IG(V): 55–158 TM: 168–190
**Hsβ4**	NM_174934	Chr 11	4489	-	214 (61)	173	229	130	3743 (94)	228	100% (100%)	IG(V): 46–151 TM: 162–183
**Mmβ4**	NM_001013390	Chr 9	4244	-	61	173	229	130	3651 (94)	228	79.4% (88.2%)	IG(V): 46–151 TM: 162–183
**Rnβ4**	NM_001008880	Chr 8	4272	-	61	173	229	130	3679 (94)	228	80.3% (88.6%)	IG(V): 46–151 TM: 162–183

Presentation and labeling as in Table 1. %ID (%SIM) refers to percentage identity and similarity with the human β4 protein sequence as determined by alignment. Zebrafish β4 subunit topology was determined as described in Methods. Numbering refers to β4 protein sequence with signal peptide intact. IG(V) domain and transmembrane regions for human β4 and human/mouse/rat splice variants were annotated based on previous reports [13, 14].

### Tissue-specific regulation of zebrafish β subunit gene expression and splicing

**Figure 5:**
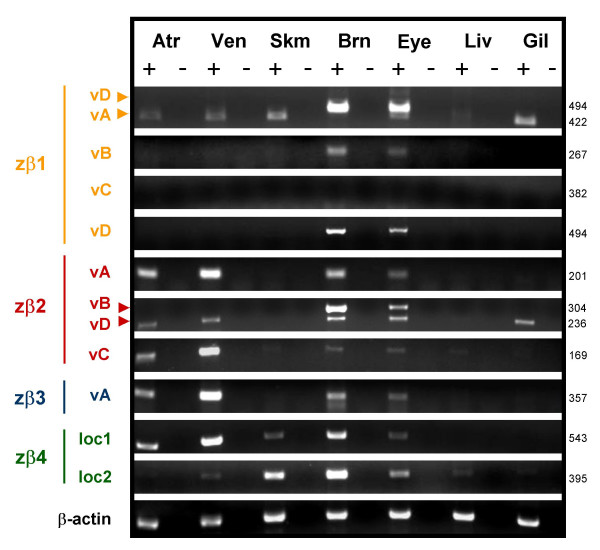
**Zebrafish sodium channel β1-4 subunit genes and novel splice variants are differentially expressed in excitable tissues**. Total RNA was isolated from wild-type adult zebrafish tissues. RT-PCR with gene and splice variant-specific primers was used to detect expression (see Table 5 for primer sequences and amplicon details). Atr = atrium, Ven = ventricle, Skm = skeletal muscle, Brn = Brain, Eye = eye/optic nerve, Liv = liver, Gil = gill. + = enzyme added to reverse transcription step, - = no reverse transcriptase enzyme (negative control). Zebrafish β-actin was amplified from each template as a positive control.

Mammalian sodium channel β subunit genes are expressed primarily in excitable tissues such as the heart, brain, and skeletal muscle. By RT-PCR, we observed that zebrafish β subunit genes are also expressed in excitable tissues where they may act to regulate the expression or function of sodium channel α subunits (Fig. 5, Table 5). Moreover, we detected distinct expression patterns for zβ1-4, suggesting that the functional importance of each β subunit likely varies by tissue type. Transcripts of zβ2 or zβ3 are absent from skeletal muscle while zβ1 and zβ4.2 are expressed only at low levels in the heart. As might be expected for non-excitable tissues, few β subunit transcripts were detected in the zebrafish liver and only zβ1 and zβ2 are expressed in the gill.

**Table 5:** Primers used to detect expression of zebrafish β subunit genes and splice variants in different tissues of the adult zebrafish.

***Forward Primer (5'-3')***	***Reverse Primer (5'-3')***	***Gene***	***Variant***	***Amplicon Size (bp) ***
CTACACTTATGCAGAAATGACAGCCAGC	GATGGACAGAGCTTCAAGCTTTTGGCT	zβ1	A	422
		zβ1	D	494
GACAGAATCCTCATCTTCCCCAACTATG	CTGCATTCTTCATTTAAACTCAGAGGT	zβ1	B	267
		zβ1	D	534
GTCCCTGCGCTGTTGTGTTTAACACAT	GGGTGAACAATCCCTTTAAGCTGCACT	zβ1	C	382
CAGCTGACAGACGAGGGCATCTACAACT	CAACACCTGCAGTGAGAAAACCCCATT	zβ2	A	201
CATCCTTGCTCTGCTCATTCTGTCCAT	TCGCTACACGATAATACCAGGGAGTGT	zβ2	B	304
		zβ2	C	169
		zβ2	D	236
CTGGTGTGTGTGGATGTGCCATCA	CTTGTTGGCGGAAAGCTTGAATGA	zβ3	-	357
AGGTGAGCACAGGGAAGGTCCATT	GGAGGCCATTTTCTGTGTTGTCGT	zβ4	loc1	543
TGTGTTGTGTTCATGCTTTG	GACCACCTTTAGTTCCTCTA	zβ4	loc2	395

Note: several primer pairs detected multiple splice variants, as indicated above and displayed in Figure 5. BP = base pairs or nucleotide number.

By designing splice variant-specific primers, we also found evidence for tissue-specific regulation of splicing of zebrafish β subunit genes (Fig. 5). While zβ1 variants B and D are expressed primarily in the brain and eye (including the optic nerve), zβ1A is expressed in the atrium, ventricle, skeletal muscle and gill. Similarly, while zβ2 variant B is primarily expressed in the brain and eye, zβ2 variants A, C, and D are also expressed in the atrium, ventricle, brain, eye, and variant D is additionally expressed in the gill. Interestingly, zβ3 may be alternatively-spliced in the brain and eye but not in the atrium or ventricle. We were unable to recover the shorter variant because of its apparent low level of expression. Although zβ1 variant C was identified by RACE-PCR using total embryonic RNA as template, we were unable to detect expression of this short splice variant in the adult tissues analyzed for this study. This suggests that zβ1 variant C may be developmentally regulated, expressed in other tissues, unstable, or is the result of an infrequent alternative splicing event.

### Zebrafish β1 functionally modifies sodium channel expression, function *in vitro*

**Figure 6:**
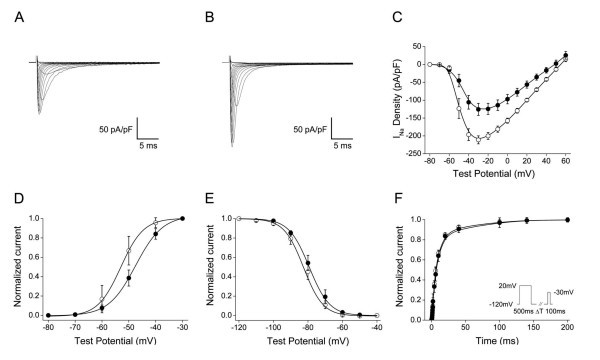
**The zebrafish β1 subunit modulates the biophysical properties of the zebrafish sodium channel α subunit zNa_v_1.5 in CHO cells**. *A) *Typical whole-cell sodium current trace of zNa_v_1.5 following expression of the pBK-CMV-*zscn5a *expression vector in CHO cells (n = 8). *B) *Typical whole-cell sodium current trace of zNa_v_1.5 + zβ1 (variant D) following co-expression of pBK-CMV-*zscn5a *and pGFP-IRES-*zβ1D*. zβ1 significantly increased the peak amplitude of sodium current by 68% (p = 0.005) at a -30 millivolt (mV) depolarizing pulse (n = 5). *C) *Current-voltage relationship demonstrating an increase in sodium current at every test potential between -50 and +50 mV. Filled circles = zNa_v_1.5 alone; open circles = zNa_v_1.5 + zβ1. *D) *Voltage dependence of activation (zNa_v_1.5 alone, n = 8; zNa_v_1.5 + zβ1, n = 5). *E) *Voltage-dependence of inactivation. (zNa_v_1.5 alone, n = 5; zNa_v_1.5 + zβ1, n = 6). F) Recovery from inactivation (zNa_v_1.5 alone, n = 4; zNa_v_1.5 + zβ1, n = 6). Pulse protocol in inset. Summary data is reported in table 6.

As the first sodium channel auxiliary subunit to be identified, β1 is the most widely-studied of the four known mammalian β subunit proteins. To evaluate whether functional α and β subunit interactions are likely to occur in zebrafish, we heterologously co-expressed the most conserved variant of *zβ1 (variant D) *with the gene encoding the zebrafish pore-forming sodium channel α subunit zNa_v_1.5, whose expression we detected in the adult zebrafish brain and heart (unpublished observations). Prior studies suggest that the mammalian β1 subunit may influence the current amplitude and possibly the gating of mammalian Na_v_1.5 *in vitro *[54-57]. Co-expression of *zβ1 *with *zscn5a *in Chinese Hamster Ovary (CHO) cells increased peak sodium current by 68% at a -30 mV depolarizing pulse (n = 5, p < 0.001) compared to *zscn5a *alone (n = 8) (Fig. 6A–C, table 6). Co-expression of *zβ1 *with *zscn5a *also resulted in small but significant changes in the gating of the zNa_v_1.5 channel protein. zβ1 induced hyperpolarizing shifts in both the voltage-dependence of activation and inactivation of zNa_v_1.5, without altering recovery from inactivation (Fig. 6D–F, table 6). These data suggest that the canonical modulatory effects of mammalian sodium channel β subunits on α subunit function and expression are likely to be conserved in teleosts and other non-mammalian vertebrates.

**Table 6:** Biophysical properties of zNa_v_**1.5 and zNa**_v_**1.5 plus zβ1 (variant D) in CHO cells**.

	Peak amplitude (pA)*	**Activation V**_1/2_**(mV)**	**Inactivation V**_1/2_**(mV)**	**Recovery from inactivation, Tau (ms)**
**zNa**_v_**1.5 alone**	-125.7 ± 16.9 pA/pF (n = 8)	-47.5 ± 1.6 mV (n = 8)	-79.3 ± 1.0 mV (n = 5)	127.9 ± 8.1 ms (n = 4)
**zNa**_v_**1.5 + zβ1**	-211.1 ± 11.2 pA/pF† (n = 5)	-53.0 ± 2.5 mV† (n = 5)	-82.6 ± 1.1 mV† (n = 6)	125.7 ± 4.1 ms (n = 6)

All values are mean ± standard error of the mean (SEM). * at a -30 mV depolarizing pulse. † p < 0.001 vs. zNa_v_1.5 alone, Student's T-Test. pA = picoamperes; mV = millivolts; ms = milliseconds; pF = picofahrads; V_1/2 _= half maximal voltage.

### Evolutionary relationships of vertebrate β subunit genes

**Figure 7:**
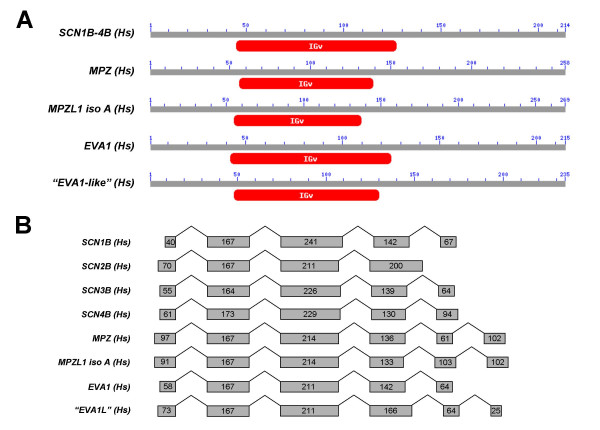
**Four additional human genes share homology with β subunits in both sequence and genomic organization**. *A) *BLASTP searches of the human genome using sodium channel β subunit amino acid sequences identified significant homology to myelin P_0 _protein (*MPZ*), myelin P_0 _protein-like protein isoform A (*MPZL1 isoform A)*, epithelial V-like antigen 1 (*EVA1*), and epithelial V-like antigen 1-like gene (*EVA1L*), all of which are single transmembrane proteins with extracellular V-type immunoglobulin domains and intracellular C-terminal tails as predicted by NCBI *conserved domain database v2.09 and TMPred *(see Methods for further details). *B) *This group of genes additionally shares similar genomic organization. Numbers in grey boxes refer to exon size (nucleotides) with untranslated sequence excluded for clarity.

Although β subunit genes appear to be related both structurally and functionally to each other and to cell adhesion molecules with V-type IG domains such as myelin protein zero and contactin [11,12], the evolutionary relationships among these genes have not previously been formally studied. To investigate these relationships, we first identified an extended group of human genes that display sequence homology to β subunits. BLAST searches of the human genome with human β subunit nucleotide and amino acid sequences most frequently-identified the genes *myelin protein zero*, *myelin protein zero-like 1, isoform A *(*MPZL1 iso A*), *epithelial V-like antigen *(*EVA1*), and hypothetical protein LOC196264 which we named "*EVA1-like gene*" (*EVA1L*) for its similarity to *EVA1 *and the proximity of these two genes in the human genome. Analysis of the amino acid sequence of each gene using the NCBI conserved domain database and TMpred software revealed that all 4 proteins likely possess single V-type IG domains and transmembrane segments, respectively, similar to sodium channel β subunits (Fig. 7A). Moreover, comparison of the complementary DNA sequences of these genes against their respective genomic loci indicated that all 4 genes also possess genomic organization similar to sodium channel β subunits (Fig. 7B). We therefore included these genes in our analysis of the synteny and phylogeny of the extended family of vertebrate β subunit genes.

**Figure 8:**
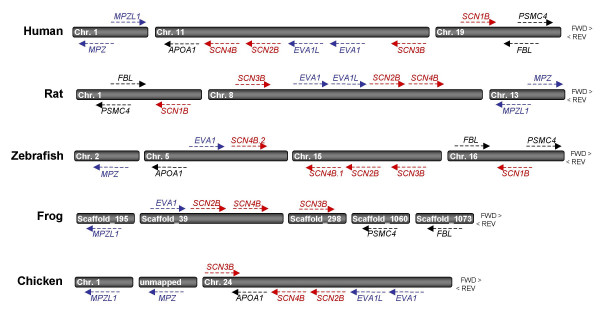
***SCN2B, SCN3B *and *SCN4B *are syntenic with each other and with *EVA1, EVA-1L*, and *APOA-1 *in multiple vertebrate genomes, while *SCN1B *is syntenic with *FBL *and *PSMC4 *in humans, rats, and zebrafish**. To analyze synteny, β subunits and subunit-like genes were identified in human, rat, zebrafish, frog and bird genomes (see Table 7 for gene IDs and physical locations). While humans and rats have four and zebrafish have five β subunit genes, only three β subunit genes were identified in frogs and birds (β1 was not found in either genome). Reciprocal blast searches and *in silico *chromosome walking were used to assess physical relationships among vertebrate β subunit-like genes. HUGO gene nomenclature symbol IDs: *PSMC4 *= 26S protease regulatory subunit 6B; *FBL *= fibrillarin; *APOA1 *= apolipoprotein A-1; *MPZ *= myelin protein zero; *MPZL1 *= myelin protein zero-like gene, isoform A; *EVA1 *= epithelial V-like antigen 1; *EVA1L *= unannotated gene similar to EVA1. Red = β subunit genes, Blue = β subunit-like genes, Black = unrelated genes that are syntenic with β subunits.

To analyze synteny, we first identified all β subunit-like genes in human, rat, zebrafish, frog (*Xenopus tropicalis*) and bird (*Gallus gallus*) genomes (Table 7). Notably, while humans and rats each have 4 and zebrafish have 5 β subunit genes, only 3 β subunit genes were identified in frogs and birds (β1 was not found in either genome). Next, we used reciprocal BLAST searches and *in silico *chromosome walking to assess physical relationships among β-subunit like genes (Fig. 8). Strikingly, *scn2b*, *scn4b*, and *EVA1/EVA1L *genes map to common locations in every vertebrate genome analyzed (human chromosome 11, rat chromosome 8, zebrafish chromosome 5/15, frog scaffold_39, and chicken chromosome 24), suggestive of a close evolutionary relationship that may have resulted from gene duplication. Although physically more distant, *scn3b *is also syntenic with this group of genes in 4/5 genomes analyzed. Of the 4 sodium channel β subunits, only *scn1b *falls outside this syntenic block. The common ancestry of human, rat, and zebrafish *scn1b *is supported, however, by the proximity of the genes encoding the 26S protease regulatory subunit 6B (*PSMC4*) and fibrillarin (*FBL*) to the *scn1b *locus in all three species (human chromosome 19, rat chromosome 1, zebrafish chromosome 16). The synteny of β subunit genes in mammalian, fish, amphibian, and avian lineages thus supports the hypothesis that extant vertebrate β subunits are orthologous. Moreover, these findings strongly suggest that the evolution of at least several members of this extended gene family (*scn2b*, *scn4b*, *EVA1*, *EVA1L*) may have arisen from duplication events that predated the divergence of teleosts and tetrapods.

**Table 7:** List of cloned and predicted genes utilized for analysis of synteny and phylogeny of the extended β subunit gene family in vertebrates.

		**Analysis of Synteny**		**Analysis of Phylogeny**
**Gene**	**Species**	**Accession No. (Gene)**	**Physical Location**	**Accession No. (Protein)**
***SCN1B***	*Homo sapiens*	ENSG00000105711	chr. 19 (FWD, 40.21–40.22 Mb)	ENSP00000262631
	*Rattus norvegicus*	ENSRNOG00000021102	chr. 1 (REV, 86.16–86.17 Mb)	n/a
	*Danio rerio*	ENSDARG00000060222	chr. 16 (REV, 47.82–47.83 Mb)	ABF47239
***SCN2B***	*Homo sapiens*	ENSG00000149575	chr. 11 (REV, 117.54–117.55 Mb)	ENSP00000278947
	*Rattus norvegicus*	ENSRNOG00000016221	chr. 8 (FWD, 48.07–48.08 Mb)	n/a
	*Danio rerio*	ENSDARG00000041176	chr. 15 (REV, 27.32 Mb)	ABF47241.
	*Xenopus tropicalis*	ENSXETESTG00000009930	*scaffold_39 (FWD, 0.58–0.59 Mb)	ENSXETESTP00000016971
	*Gallus gallus*	ENSGALG00000021272	chr. 24 (REV, 5.10–5.11 Mb)	ENSGALP00000033652
***SCN3B***	*Homo sapiens*	ENSG00000166257	chr. 11 (REV, 123.01–123.03 Mb)	ENSP00000299333
	*Rattus norvegicus*	ENSRNOG00000006937	chr. 8 (FWD, 43.23–43.25 Mb)	n/a
	*Danio rerio*	ENSDARESTG00000011553	chr. 15 (REV, 28.62–28.64 Mb)	ABF47244
	*Xenopus tropicalis*	ENSXETESTG00000002589	*scaffold_298 (FWD, 0.94–0.96 Mb)	ENSXETESTP00000004357
	*Gallus gallus*	ENSGALESTG00000012587	chr. 24 (FWD, 2.82–2.83 Mb)	ENSGALESTP00000019882
***SCN4B.1***	*Homo sapiens*	ENSG00000177098	chr. 11 (REV, 117.51–117.53 Mb)	ENSP00000322460
	*Rattus norvegicus*	ENSRNOG00000026679	chr. 8 (FWD, 48.09–48.11 Mb)	n/a
	*Danio rerio*	ENSDARESTG00000010162	chr. 15 (REV, 27.26–27.29 Mb)	ABF47245
	*Xenopus tropicalis*	ENSXETESTG00000009931	*scaffold_39 (FWD, 0.61–0.63 Mb)	ENSXETESTP00000016972
	*Gallus gallus*	ENSGALG00000007409	chr. 24 (REV, 5.10 Mb)	ENSGALP00000011971
***SCN4B.2***	*Danio rerio*	ENSDARESTG00000000878	chr. 5 (FWD, 38.08–38.10 Mb)	ABF47246
***MPZ***	*Homo sapiens*	ENSG00000158887	chr. 1 (REV, 159.54–159.55 Mb)	ENSP00000289928
	*Rattus norvegicus*	ENSRNOESTG00000011377	chr. 13 (FWD, 87.04–87.05 Mb)	n/a
	*Danio rerio*	ENSDARG00000038609	chr. 2 (REV, 46.60–46.62 Mb)	ENSDARP00000056371
	*Gallus gallus*	ENSGALESTG00000015635	*chr. Un (REV, 60.79–60.80 Mb)	ENSGALP00000008810
***MPZL1 isoA***	*Homo sapiens*	ENSG00000197965	chr. 1 (FWD, 165.96–166.03 Mb)	ENSP00000352513
	*Rattus norvegicus*	ENSRNOG00000003248	chr. 13 (REV, 81.32–81.36 Mb)	n/a
	*Xenopus tropicalis*	ENSXETG00000023014	*scaffold_195 (REV, 1.78–1.79 Mb)	ENSXETP00000049765
	*Gallus gallus*	ENSGALG00000015438	chr. 1 (REV, 85.41–85.44 Mb)	ENSGALP00000024855
***EVA1***	*Homo sapiens*	ENSG00000149573	chr. 11 (REV, 117.63–117.64 Mb)	ENSP00000278937
	*Rattus norvegicus*	ENSRNOG00000016085	chr. 8 (FWD, 47.99–48.00 Mb)	n/a
	*Danio rerio*	ENSDARG00000027345	chr. 5 (FWD, 38.05–38.07 Mb)	ENSDARP00000032601
	*Xenopus tropicalis*	ENSXETESTG00000008504	*scaffold_39 (FWD, 0.51–0.54 Mb)	ENSXETESTP00000014524
	*Gallus gallus*	ENSGALG00000007412	chr. 24 (REV, 5.12–5.13 Mb)	ENSGALP00000011976
***"EVA1-like"***	*Homo sapiens*	ENSG00000160588	chr. 11 (REV, 117.60–117.63 Mb)	ENSP00000278949
	*Rattus norvegicus*	ENSRNOG00000026753	chr. 8 (FWD, 48.00–48.01 Mb)	n/a
	*Gallus gallus*	ENSGALG00000021271	chr. 24 (REV, 5.11–5.12 Mb)	ENSGALP00000033649
***PSMC4***	*Homo sapiens*	ENSG00000013275	chr. 19 (FWD, 45.17–45.18 Mb)	n/a
	*Rattus norvegicus*	ENSRNOG00000018994	chr. 1 (REV, 83.15–83.16 Mb)	n/a
	*Danio rerio*	ENSDARG00000027099	chr. 16 (FWD, 47.99-5-48.00 Mb)	n/a
	*Xenopus tropicalis*	ENSXETG00000014266	*scaffold_1060 (REV, 0.17 Mb)	n/a
***FBL***	*Homo sapiens*	ENSG00000105202	chr. 19 (REV, 45.02–45.03 Mb)	n/a
	*Rattus norvegicus*	ENSRNOG00000019229	chr. 1 (FWD, 83.27–83.28 Mb)	n/a
	*Danio rerio*	ENSDARG00000053912	chr. 16 (FWD, 47.49–47.50 Mb)	n/a
	*Xenopus tropicalis*	ENSXETG00000017830	*scaffold_1073 (REV, 0.14 Mb)	n/a
***APOA1***	*Homo sapiens*	ENSG00000118137	chr. 11 (REV, 116.21 Mb)	n/a
	*Danio rerio*	ENSDARG00000012076	chr. 5 (REV, 38.04 Mb)	n/a
	*Gallus gallus*	ENSGALG00000007114	chr. 24 (REV, 4.79–4.80 Mb)	n/a

All genes and proteins are identified by Ensembl accession numbers, except for cloned zebrafish beta subunit protein sequences (which begin with ABF and represent accession numbers assigned by GenBank). * = unmapped scaffolds in Ensembl database. FWD = forward and REV = reverse and refers to gene orientation. n/a = not applicable (e.g. proteins for this species were not included in phylogenetic analysis).

**Figure 9:**
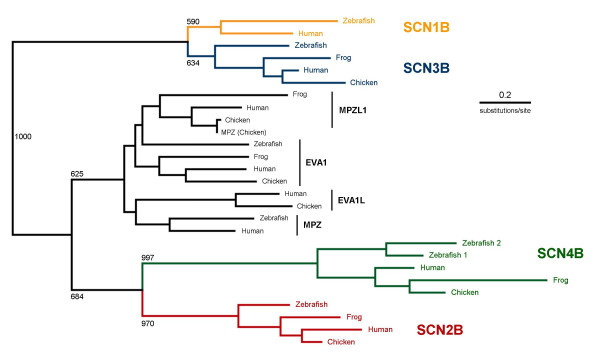
**Phylogenetic analysis demonstrates that vertebrate sodium channel β1-4 subunit genes are orthologous, that β1/β3 and β2/β4 are closely related, and that zβ4.1 and zβ4.2 resulted from a recent gene duplication in fish**. Actual (human, zebrafish) and predicted (chicken, frog) amino acid sequences of β subunit and related genes were aligned using CLUSTALX (v1.83). Phylogenetic trees were reconstructed using the neighbor-joining method of Saitou and Nei and viewed with NJPlot software. Alignment gaps were excluded and the Kimura correction was made for multiple substitutions. Bootstrapping (n = 1000) was applied to test the robustness of each node. Tree is unrooted due to the lack of evidence for β subunit-like genes in invertebrate species. HUGO gene nomenclature symbol IDs: *MPZ *= myelin protein zero; *MPZL1 *= myelin protein zero-like gene isoform A; *EVA1 *= epithelial V-like antigen 1; *EVA1L *= unannotated gene similar to *EVA1*.

To further analyze the evolution of vertebrate β subunit-like genes, we reconstructed the phylogeny of this gene family (Fig. 9). Neighbor-joining phylogenetic analysis of amino acid sequences demonstrated that all-identified sodium channel β subunits fall on one of four branches corresponding to β1, β2, β3, or β4, supporting the preliminary identity assigned to each cloned zebrafish β subunit gene (including the duplicated zebrafish β4 genes) by alignment with each individual mammalian subunit (Figs. 1A, 2A, 3A, 4A). Despite synteny among *scn2b*, *scn3b*, and *scn4b*, phylogenetic analysis revealed that β3 is more closely related to β1 than to either β2 or β4. Moreover, our phylogenetic model incorporating β subunit-like genes additionally indicated that β2 and β4 are more closely-related to each other and to β subunit-like genes than to either β1 or β3. Our analysis thus supports an evolutionary model where β2/β4/β subunit-like genes and β1/β3 arose following duplications of 2 distinct precursor genes. The timing of such duplications cannot be gleaned from this model. The presence of gene orthologs to all 4 β subunits in divergent vertebrates (mammals and teleosts) strongly suggests, however, that the early vertebrate common ancestor to these lineages already had four distinct β subunit genes. Duplication events that occurred in this gene family – aside from that which produced two zebrafish β4 genes – thus occurred earlier in vertebrate evolution or prior to the emergence of the vertebrates altogether.

### Absence of β subunit genes in invertebrates

The sequenced genomes of the ascidians *Ciona intestinalis* and *Ciona savignyi* provide an opportunity to identify putative precursors to vertebrate β subunits genes in invertebrate chordates [58,59]. Although both ascidian genomes contain several loci encoding Na_v_1 voltage-gated sodium channel α subunits, BLAST searches using the nucleotide and protein sequences of zebrafish and mammalian sodium channel β subunits and related proteins (MPZ, EVA1) did not identify any homologous genes. The absence of β subunit-like genes in sequenced Ciona genomes does not preclude the presence of numerous other genes with V-type and other IG-like domains ([60,61] and unpublished observations). These findings strongly suggest that sodium channel β subunit genes are an innovation of vertebrates. As predicted, searches of the genome of the echinoderm Strongylocentrotus purpuratus (sea urchin) [62], a non-chordate deuterostome, also did not reveal any sodium channel β subunits genes.

Despite the lack of evidence for β subunits genes outside of vertebrates, studies conducted in *Drosophila melanogaster *demonstrate that invertebrate sodium channels require additional subunits for normal function. Mutations in the *Drosophila tip-E (temperature-induced paralysis) *gene, which encodes a sodium channel auxiliary subunit, disrupt nerve conduction by perturbing the expression and function of the *para *voltage-gated sodium channel [63-67]. Moreover, 4 recently-identified *tip-E *related genes (*TEH1-4 *or *tip-E *homologs 1–4) were found to modulate the density and kinetics of sodium currents of heterologously-expressed *para *sodium channels [68]. Based on these findings, we sought to assess whether vertebrate and invertebrate sodium channel auxiliary subunits share common structural elements or functional domains. Use of TMPred software and the NCBI conserved domain database demonstrated, however, that *Drosophila *tip-E and tip-E homologous genes (*TEH1-4*) each possess two predicted membrane-spanning segments (not one) and lack the canonical V-type IG domain found in vertebrate β subunits. Additionally, alignment of the amino acid sequences of cloned zebrafish and human sodium channel β subunit genes with the sequences of *Drosophila *tip-E and tip-E homologous genes also revealed only minimal homology (< 15% amino acid identity). BLAST searches of the *Drosophila *genome did not uncover any additional genes sharing close homology with vertebrate β subunits, despite the presence of numerous genes encoding proteins with immunoglobulin domains ([69] and unpublished observations). These results indicate that vertebrate and invertebrate sodium channel auxiliary subunits are dissimilar in structure and unrelated.

## Discussion

Numerous studies of mammalian voltage-gated sodium channel β subunits have demonstrated that these small, single membrane-spanning proteins are integral components of sodium channel complexes in excitable tissues, modulators of the expression and function of pore-forming sodium channel α subunits, and candidate genes for clinical disorders linked to perturbed membrane excitability such as arrhythmia and epilepsy [18,70]. Despite the early origins of the Na_v_1 family of sodium channel α subunits and their cloning from diverse metazoans including eels, jellyfish, flies, and humans, the evolutionary history of sodium channel β subunits has remained obscure. The primary objective of this study was thus to investigate β subunit genes in *Danio rerio *(zebrafish), a pivotal vertebrate species whose teleost ancestors diverged from mammals over 400 million years ago, in order to gain further insight into the origin and regulation of the voltage-gated sodium channel macromolecular complex.

Using a combination of bioinformatics, molecular cloning, and phylogenetic analysis, we identified conserved orthologs of all 4 mammalian β subunit genes and 8 novel β1 and β2 splice variants in zebrafish. Using our cloned zebrafish sequences, we subsequently identified β subunit genes in other non-mammalian vertebrates including *Xenopus tropicalis *(Western clawed frog) and *Gallus gallus *(Red jungle fowl). The existence of conserved mammalian, teleost, amphibian, and avian sodium channel β subunit loci indicates that this gene family is likely to be found in most if not all vertebrate genomes. Moreover, our detection of zebrafish β subunit gene expression in tissues including the heart, muscle, and the brain suggests that the voltage-gated sodium channel α-β subunit macromolecular complex is likely to be a common structural feature of excitable membranes.

### Sodium channel complexes in non-mammalian vertebrates: an emerging concept

Our data are the first to support the existence of voltage-gated sodium channel α-β subunit macromolecular complexes in non-mammalian vertebrates. Data from prior studies, however, suggest that sodium channels form α-β subunit complexes in mammals but not in other vertebrate lineages [5-7,71-79]. While sodium channels biochemically purified from rat neuronal membranes and rat and rabbit skeletal muscle membranes were found to be comprised of 1 α subunit and 1 or more auxiliary β subunits, for example, α subunits purified from the electroplax (electric organ) of the South American eel *Electrophorus electricus *and from chick cardiac muscle were unaccompanied by β subunits [5-7,71-79]. Further investigation into the existence of sodium channel α-β macromolecular complexes in non-mammalian vertebrates is thus needed to reconcile our current findings with the results of previous studies.

There are several possible reasons for the discord between biochemical and molecular genetic approaches. First, it is possible that some but not all voltage-gated sodium channel isoforms within a particular species form complexes by associating with auxiliary β subunits, or that pore-forming α subunits form complexes only in specific tissues. Evidence suggests that even in mammals, for example, the subunit composition of voltage-gated sodium channels significantly varies both by α subunit isoform and by tissue [9,72]. Second, at least for the eel electroplax, the function of this organ may depend on sodium channel α subunits that have evolved unique properties which may preclude association with auxiliary subunits [80]. Third, it is possible that sodium channel α subunit isoforms in certain tissues form complexes not with canonical β subunits but with yet unidentified proteins. In mammals, the interaction of sodium channel α subunits with a number of additional components and modulators in macromolecular complexes suggest that β subunits may not always serve as obligatory functional partners (for review, see refs [18,81]). Our detection of conserved β subunit mRNA expression in excitable organs such as heart, brain, and skeletal muscle, however, suggests the strong possibility of α-β subunit complexes in these zebrafish tissues. Moreover, co-expression of zebrafish α and β subunit genes in CHO cells demonstrated meaningful functional interactions between the two subunits, with significant differences observed in sodium current amplitude and channel gating when both subunit genes are expressed together versus when the α subunit gene is expressed alone. Despite these findings, however, we cannot completely rule out the possibility that sodium channel β subunit genes play different *in vivo *functional roles in mammals than in non-mammalian vertebrates.

### Evolution of sodium channel β subunits and related genes

Our analysis of the synteny and phylogeny of vertebrate sodium channel β subunit genes suggests a different evolutionary history for α and β subunits. For sodium channel α subunits, prevailing evolutionary models posit that the 10 mammalian isoforms arose from the tandem duplication of at least 2 of 4 ancestral sodium channel genes, which in turn had duplicated from 1 or 2 precursor chordate Na_v_1 sodium channel genes by polyploidization [36-39]. As would be predicted by this model, the total number of Na_v_1 sodium channel genes varies between teleosts and mammals despite the phylogenetic clustering of all of these genes into 4 groups derived from ancestral vertebrate sodium channel genes [38,40]. Sodium channel β subunits, which are fewer in number, do not appear to have undergone tandem duplication in either mammals or in non-mammalian vertebrates. This is supported by our finding that 4 distinct vertebrate lineages (mammals, ray-finned fishes, amphibians and birds) all appear to share distinct orthologs to 3 or 4 β subunit genes. Thus, it is likely that the common ancestor to teleosts and tetrapods over 400 million years ago also possessed 4 distinct ancestral β subunit genes (β1-4). The presence of 2 β4 orthologs on different zebrafish chromosomes is likely to be the remnant of an additional polyploidization event known to have occurred in teleost vertebrates but not in mammals [41]. It is difficult to determine why duplicate genes for β1-3 have not been retained in zebrafish; it is possible that > 5 β subunit genes did not offer teleosts any evolutionary advantage [42].

The close phylogenetic relationship of *SCN1B *and *SCN3B *and the physical proximity of *SCN2B *and *SCN4B *to each other and to genes (e.g. *EVA1*) that are β subunit-like in sequence and genomic organization is strong evidence that duplication events gave rise to the β subunit gene family during the postulated large-scale expansion of the early vertebrate genome. Although we cannot accurately predict the early evolutionary history of sodium channel β subunit genes prior to the divergence of teleosts from other vertebrate lineages, the results of our phylogenetic analysis support the existence of 2 ancestral β subunit genes (β1/β3 and β2/β4) in early vertebrates. Somewhat surprisingly (but in agreement with a previous report [33]), BLASTN and BLASTP searches of available urochordate (*Ciona intestinalis*, *Ciona savignyi*) genomes did not identify any sodium channel β subunit-like genes, despite the clear existence of genes encoding proteins with immunoglobulin domains similar to those found in β subunits [60,61]. Moreover, we and others also found limited homology between vertebrate β subunit genes and the *tip-E*/*TEH *subunits of the *para *sodium channel in *Drosophila *[34,65]. These findings strongly suggest that sodium channel β subunits are a unique vertebrate innovation.

The similarity of vertebrate sodium channel β subunits to members of a more ancient family of IG domain-containing proteins suggests that β subunit genes may have arisen *de novo *in early vertebrates from genes encoding cell-adhesion molecules, membrane receptors, or components of the innate immunity. The property of modulation of the expression and function of sodium channel β subunits may thus represent a more evolutionarily recent function for this gene family. These findings support the idea that molecular mechanisms enhancing functional diversity and specialization in electrical signaling – either through α subunit gene duplication, editing or alternative splicing of α subunit RNA transcripts, or the emergence of auxiliary proteins that associate with α subunits to modulate their expression and/or function – may have been adaptive and underwent selection in the evolving nervous system of early vertebrates and subsequently, in the increasingly complex excitable tissues of diverse vertebrate lineages including mammals. Despite the lack of common ancestry among invertebrate *tip-E/TEH *subunits and vertebrate IG-like β subunits, the apparent independent evolution of Na_v_1 sodium channel-interacting proteins in distantly-related species demonstrates that the formation of sodium channel macromolecular complexes is an evolutionary advantageous and conserved mechanism for fine-tuning the properties of excitable membranes.

### Functional implications of alternative splicing of zebrafish β subunit genes

The evidence we present for the extensive C-terminal alternative splicing of zebrafish β subunit genes is intriguing because it may represent another conserved molecular mechanism for modulating electrical signaling in vertebrates. Alternative splicing has been reported for both mammalian sodium channel α and β subunits and invertebrate α subunits, often with important functional consequences [49,50,82-88]. The intronic retention events that produce both the rat β1A and human β1B variants each result in a protein with a novel transmembrane domain and intracellular C-terminal tail. When stably expressed in Chinese Hamster Lung (CHL) fibroblasts, rat β1 and β1A differentially modulate the function of the rat sodium channel α subunit Na_v_1.2 (*scn2a*) [49]. Similarly, the human β1 and β1B subunits differentially modulate the function of human Na_v_1.2 in *Xenopus *oocytes [50]. These studies suggest that the transmembrane domain and C-terminus of the human and rat β1 subunits contribute to the functional modulation of sodium channel α subunits. In a subsequent study, the β1 intracellular C-terminal tail in particular was found to be required for both efficient physical association with Na_v_1.2 and for the modulation of sodium channel function in both mammalian cells and *Xenopus *oocytes [89]. This raises the possibility that the zebrafish β1 and β2 subunit splice variants identified in this study may also differentially influence the function of sodium channel α subunits. Our identification of distinctive expression patterns for different splice variants of the same gene underscores the complexity of this putative sodium channel regulatory mechanism.

## Conclusion

We have identified conserved orthologs to all 4 mammalian β subunit genes (*zβ1-zβ4*) in zebrafish. Despite highly-conserved genomic organization in fish and mammals, zebrafish express 8 distinct mRNA transcripts for the β1 and β2 genes that are generated by alternative splicing. Zebrafish β subunit genes and their splice variants are differentially-expressed in excitable tissues, and co-expression of *zβ1 (variant D) *with the zebrafish sodium channel α subunit gene z*scn5a *in CHO cells demonstrated functional α-β interactions that we predict may also occur in native tissues. Our evolutionary analysis of mammalian, teleost, amphibian, and avian β subunit and related genes indicated that all extant vertebrate β subunits are orthologous, that β2/β4 and β1/β3 share common ancestry, and that β subunits are closely-related to other proteins with V-type IG domains including myelin protein zero (MPZ) and epithelial V-like antigen 1 (EVA1). Homologs to vertebrate β subunit genes were not identified in the genomes of invertebrate chordates, and β subunits were found to be unrelated to the *tip-E/TEH *subunits of the *para *sodium channel in *Drosophila*. Taken together, these findings suggest that the family of sodium channel β subunit genes emerged early in vertebrate evolution, prior to the divergence of teleosts and tetrapods. The evolutionary history of vertebrate β subunits is thus consistent with the hypothesis that voltage-gated sodium channel complexes are evolutionarily-conserved structural entities, and that β subunit genes may have played a role in the functional diversification and specialization of electrical signaling in early vertebrates.

## Methods

### Identification and cloning of *zSCN1B-4B* genes and splice variants

Mammalian *SCN1B-4B *gene sequences were used in BLASTN queries of the draft zebrafish genome (Ensembl Zv2-5) to identify DNA contigs containing putative zebrafish orthologs. Primers directed against predicted exons were then used to amplify partial β subunit gene sequences using the Titan RT-PCR enzyme system (Roche) and total day 2 embryonic zebrafish RNA as template. RNA was isolated using Trizol (GIBCO), digested with RQ1 DNase (Promega), and purified with the RNeasy Mini Kit (Qiagen). 5' and 3' ends and alternatively-spliced forms of each gene were identified using 5' and 3' RNA ligase mediated rapid amplification of cDNA ends (RLM-RACE) PCR (Ambion). Additional splice variants were identified by RT-PCR while examining tissue-specific expression. All amplicons were subcloned directly into the pGEM-TEasy vector (Promega) and sequenced in their entirety. Consensus sequences for each gene were established after comparing the forward and reverse sequences of a minimum of 5 clones.

### Sequence data and annotation

Eleven novel zebrafish gene sequences were deposited in the NCBI GenBank database: zβ1A [Genbank:DQ489722], zβ1B [Genbank:DQ489723], zβ1C [Genbank:DQ489724], zβ1D [Genbank:DQ489725], zβ2A [Genbank:DQ489726], zβ2B [Genbank:DQ489727], zβ2C [Genbank:DQ489728], zβ2D [Genbank:DQ489729], zβ3 [Genbank:DQ489730], zβ4 locus 1 [Genbank:DQ489731], zβ4 locus 2 [Genbank:DQ489732]. Intron-exon boundaries for each gene and splice variant were determined by comparing cDNA and genomic sequences. Sequences for zebrafish β1-β4 were annotated by alignment with their mammalian orthologs using the AlignX function of Vector NTI v9.1 (Invitrogen) and by use of the following informatics resources: signal peptide (SMART) [90], IG-like domain (SMART [90], NCBI conserved domain database, CDD, v2.06 [91], and REF [14]), transmembrane (TMpred [92] and REF [93]), disulfide bridge (Prosite) [94], and putative N-linked glycosylation sites (NetNGlyc 1.0 server) [95].

### Gene expression

Tissues were dissected from wild type adult zebrafish (strain TuAB, 12–16 months old) and flash frozen on dry ice in ethanol. Tissue-specific total RNA was isolated and purified as described above. First strand cDNA synthesis was performed using 1 μg of RNA from each tissue, random hexamer primers, and Transcriptor reverse transcriptase enzyme (Roche). 2 μl of first strand cDNA was used in 25 μl PCR reactions with Expand High Fidelity DNA polymerase (Roche). Amplicons were analyzed on a 1–2% agarose gel made with 1 x Tris-Acetate-EDTA (TAE) buffer. Primer pairs used to amplify each individual gene and splice variant are listed in Table 5. All amplicons were subcloned directly into the pGEM-TEasy vector (Promega) and sequenced in their entirety in both sense and antisense directions.

### Identification of β subunit-like genes

Human sodium channel β subunit nucleotide and amino acid sequences were used in BLAST searches of the human genome to identify β subunit-like genes. Four human genes sharing the greatest homology as well as similar genomic organization and/or synteny with known sodium channel β subunit genes were included for further analysis: myelin protein zero (MPZ) [Genbank:NP_000521], myelin protein zero-like gene, isoform A (MPZL1 isoform A) [Genbank:NP_003944], epithelial V-like antigen (EVA) [Genbank:NP_005788], and epithelial V-like antigen-like gene (hypothetical protein LOC196264 or EVA1-like gene) [Genbank:NP_938016].

### Analysis of synteny

Synteny between vertebrate β subunits and related genes was assessed by chromosomal walking and reciprocal BLAST searches of genes adjacent to β subunit loci in human, zebrafish, *Rattus norvegicus*, *Xenopus tropicalis*, and *Gallus gallus *genome databases (Ensembl).

### Analysis of phylogeny

To estimate phylogeny, additional β subunit and β subunit-like gene sequences were identified in *Rattus norvegicus*, *Xenopus tropicalis*, and *Gallus gallus *genome databases (Ensembl). Predicted amino acid sequences were aligned with cloned zebrafish sequences using CLUSTALX (v1.83). Phylogenetic trees were reconstructed using the neighbor-joining method of Saitou and Nei (Ref [96]) and viewed with NJplot software. Alignment gaps were excluded in the analysis, and the Kimura correction was made for multiple substitutions [97]. The robustness of each node in the phylogenetic tree was analyzed with bootstrap analysis (n = 1000). Trees were unrooted due to limited evidence for β subunit-like genes in non-vertebrate species.

### Genome databases

All genome resources utilized for this study were accessed through the Ensembl gateway [98] with the exception of the sea urchin genome, which is available for BLAST searches by the National Human Genome Sequencing Center at Baylor College of Medicine [99].

### Expression vectors

The full-length zebrafish β1D splice variant was amplified in one step from total D2 embryonic RNA template using the Titan one-tube RT-PCR system (Roche) and the following primers: Fwd: *Spe I*-GACTCTGAAAACAAAGCCTG, Rev: *Sac II*-AGAGCTTCAAGCTTTTGGCT. The 733 base pair product was sublconed directly into the pGEM-TEasy vector. Multiple clones were purified, sequenced, and screened against the consensus sequence determined during the cloning of the zβ1 gene. Insert was digested out of a single zβ1D consensus clone and subcloned into the pGFP-IRES vector (Clontech) using *Spe I *and *Sac II *restriction sites. We have used similar methods to identify, clone and assemble a full-length *zscn5a *expression construct (pBK-CMV-*zscn5a*) which encodes zebrafish Na_v_1.5. These methods are described elsewhere (manuscript in preparation).

### Transient transfection and electrophysiology

Cultured Chinese Hamster Ovary (CHO) cells were transiently transfected with pBK-CMV-*zscn5a *and pGFP-IRES-*zβ1D *constructs using FuGENE6 (Roche). Cells were grown for 48 hours after transfection before electrophysiologic study. Whole-cell voltage clamp was performed at room temperature with 2-MΩ patch microelectrodes and an Axopatch 200A amplifier. To minimize the capacitive transients, we compensated for approximately 70% to 80% of the cell capacitance and series resistance [100]. Cells exhibiting very large currents (> 6 nA) were also excluded from further analysis. Cells were also excluded if voltage control was compromised. The extracellular bath solution contained (in mmol/L) NaCl 145, KCl 4.0, MgCl_2 _1.0, CaCl_2 _1.8, glucose 10, and HEPES 10; the pH was 7.4, adjusted with NaOH. The pipette (intracellular) solution contained (in mmol/L) NaF 10, CsF 110, CsCl 20, EGTA 10, and HEPES 10; the pH was 7.4, adjusted with CsOH. Cells were held at -120 mV, and activating currents were elicited with depolarizing pulses from -100 to +50 mV in 10 mV increments. Specific clamp protocols are indicated with the data. Data were acquired by pClamp8.0 (Axon Instruments Inc), sampled at 50 kHz, and low-pass filtered at 5 kHz. All currents were normalized to the cell capacitance calculated by Membrane Test (OUT O) in pClamp8.0.

## Abbreviations

BLAST Basic local alignment search tool

BLASTN BLAST nucleotide

BLASTP BLAST protein

dbSNP Database of single nucleotide polymorphisms

HUGO Human genome organization

IRES Internal ribosomal entry site

mRNA Message ribonucleic acid

Na_v_1 Voltage-gated sodium channel, family 1

NCBI National Center for Biotechnology Information

*Para Paralytic *gene, *Drosophila*

PCR Polymerase chain reaction

PharmGKB Pharmacogenomics knowledge base

RACE Rapid amplification of cDNA ends

RT-PCR Reverse transcription polymerase chain reaction

SNPs Single nucleotide polymorphisms

*TEH1-4Tip-E *homologous genes 1–4

*Tip-ETemperature-induced paralysis *gene, *Drosophila*

TMPred Transmembrane prediction software

UTR Untranslated region

*zβ1 *zebrafish sodium channel beta 1 subunit gene (zebrafish *scn1b*).

*zβ2 *zebrafish sodium channel beta 2 subunit gene (zebrafish *scn2b*).

*zβ3 *zebrafish sodium channel beta 3 subunit gene (zebrafish *scn3b*).

*zβ4.1 *zebrafish sodium channel beta 4.1 subunit gene (zebrafish *scn4.1b*).

*zβ4.2 *zebrafish sodium channel beta 4.2 subunit gene (zebrafish *scn4.2b*).

z*scn5a *zebrafish homolog to the mammalian sodium channel pore-forming α subunit gene *scn5a*.

## Authors' contributions

SSC designed this study with input from DMR and TPZ. SSC collected, analyzed, and summarized the data for all figures and tables except Figure 6/Table 6, which represent electrophysiological analyses performed by HW using expression constructs built by SSC. DMR and TPZ were the co-principal investigators of this project, overseeing the design, interpretation, and reporting of this study. SSC wrote the manuscript and all authors read and approved of the final version.

## Supplementary Material

Additional file 1 **Hydropathy analysis of zebrafish β subunit amino acid sequences reveals conserved protein secondary structure**. TMPred hyropathy plot based on the method of Kyte and Doolittle. Dotted red line drawn at 0 (neutral). SP = signal peptide and TM = transmembrane domain. Dotted line plot (black) indicates outside > inside orientation relative to membrane, while solid line plot (black) indicates inside > outside orientation. ***A) ***Hydropathy plots for zβ1 variants A, B, and D are nearly identical (zβ1D shown). ***B) ***zβ2A and zβ2B-D. ***C) ***zβ3. ***D) ***zβ4.1 and zβ4.2. Click here for file

Additional file 2 **Prediction of conserved extracellular V-type IG domains in the amino acid sequences of the most highly-conserved splice variants of zβ1-4, NCBI Conserved Domain Database (CDD *v2.09***. Predicted V-type IG domain is shown in red, and the scale denotes the length of the full-length protein. ***A) ***zβ1D, ***B) ***zβ2B, ***C) ***zβ3, ***D) ***zβ4.1, ***E) ***zβ4.2. Click here for file
