# Supplementary figures and images for: Molecular cloning and analysis of zebrafish voltage-gated sodium channel beta subunit genes: implications for the evolution of electrical signaling in vertebrates
Source: BMC Evol Biol. 2007 Jul 10;7:113. doi: 10.1186/1471-2148-7-113 (PMC1971062; doi:10.1186/1471-2148-7-113)

## Slide 1
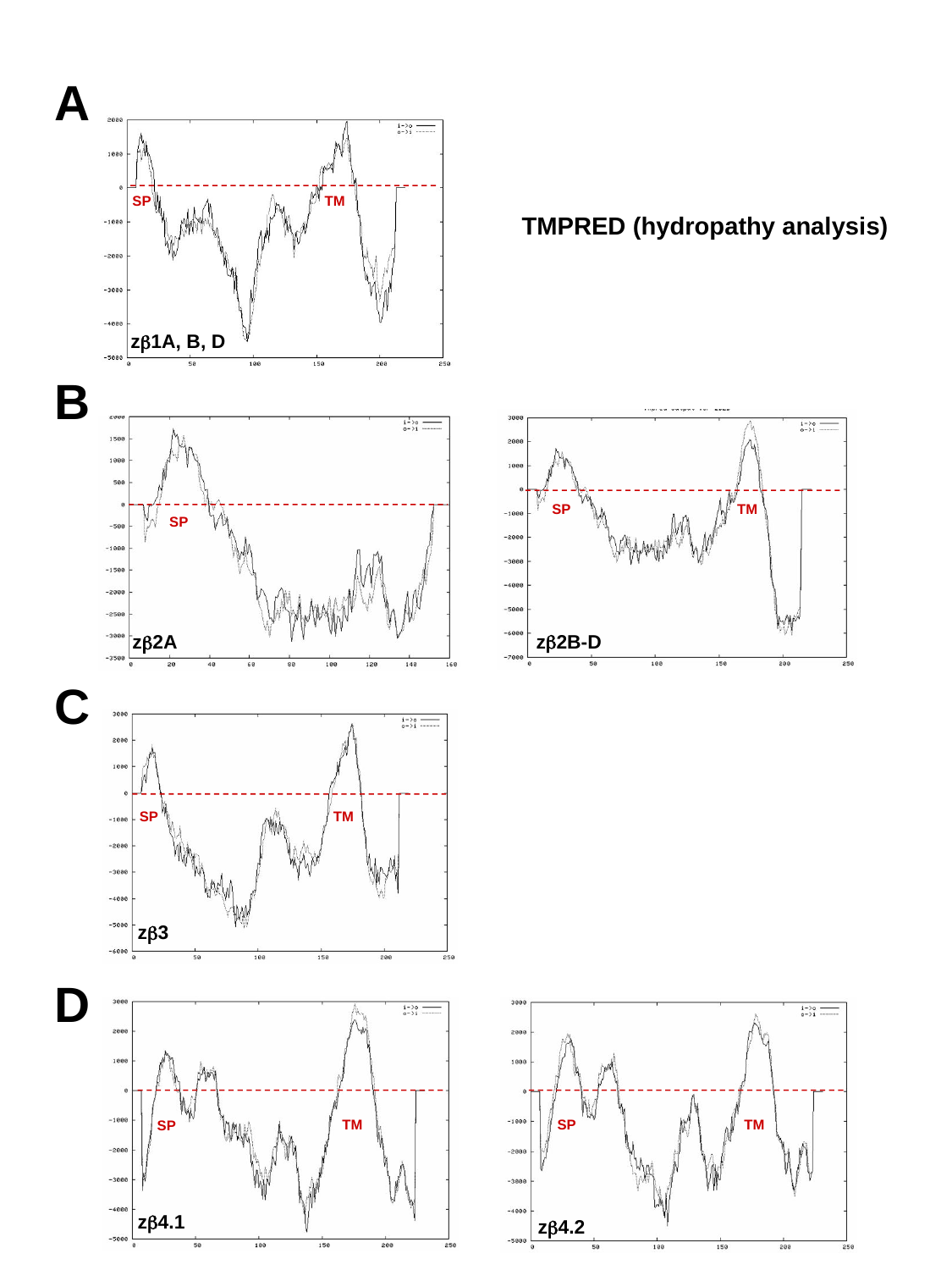

A
SP
TM
zβ1A, B, D
TMPRED (hydropathy analysis)
B
SP
TM
zβ2B-D
SP
zβ2A
C
SP
TM
zβ3
D
TM
SP
TM
SP
zβ4.1
zβ4.2

Supplement: Additional file 1 — Hydropathy analysis of zebrafish β subunit amino acid sequences reveals conserved protein secondary structure. TMPred hyropathy plot based on the method of Kyte and Doolittle. Dotted red line drawn at 0 (neutral). SP = signal peptide and TM = transmembrane domain. Dotted line plot (black) indicates outside > inside orientation relative to membrane, while solid line plot (black) indicates inside > outside orientation. A) Hydropathy plots for zβ1 variants A, B, and D are nearly identical (zβ1D shown). B) zβ2A and zβ2B-D. C) zβ3. D) zβ4.1 and zβ4.2. [file 1471-2148-7-113-S1.ppt]

## Slide 1
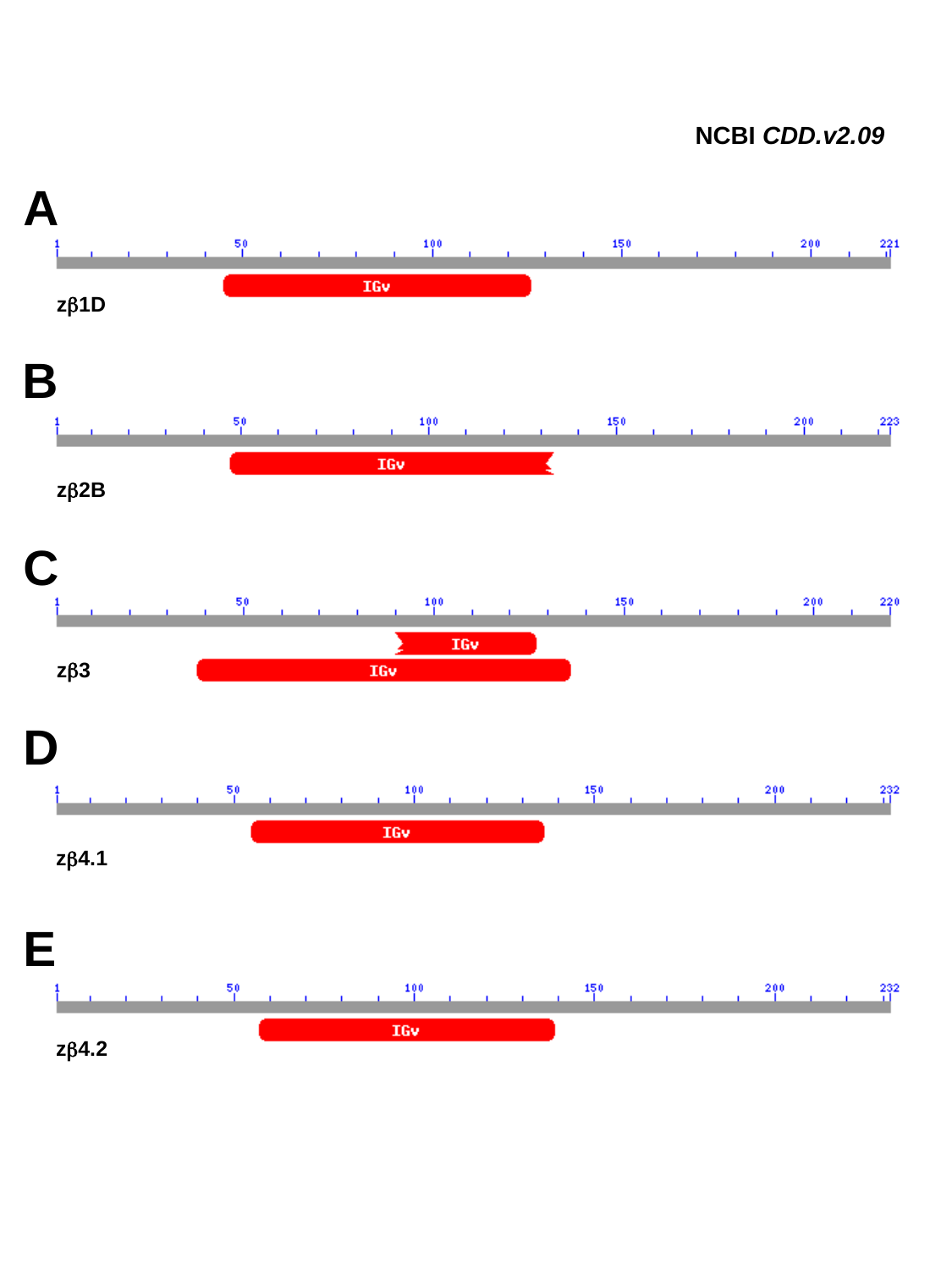

NCBI CDD.v2.09
A
zβ1D
B
zβ2B
C
zβ3
D
zβ4.1
E
zβ4.2

Supplement: Additional file 2 — Prediction of conserved extracellular V-type IG domains in the amino acid sequences of the most highly-conserved splice variants of zβ1-4, NCBI Conserved Domain Database (CDD v2.09. Predicted V-type IG domain is shown in red, and the scale denotes the length of the full-length protein. A) zβ1D, B) zβ2B, C) zβ3, D) zβ4.1, E) zβ4.2. [file 1471-2148-7-113-S2.ppt]
